# Lower serum cystatin C level predicts poor functional outcome in patients with hypertensive intracerebral hemorrhage independent of renal function

**DOI:** 10.1111/jch.14609

**Published:** 2022-12-22

**Authors:** Yongfang Zhou, Wentao Dong, Likun Wang, Siying Ren, Weiqing Wei, Guofeng Wu

**Affiliations:** ^1^ Second Affiliated Hospital of Soochow University Suzhou China; ^2^ Department of Emergency Affiliated Hospital of Guizhou Medical University Guiyang China

**Keywords:** cystatin C, estimated glomerular filtration rate, functional outcome, hypertensive intracerebral hemorrhage, neuroprotective effect

## Abstract

We explored the association between the serum level of cystatin C (CysC) at admission and short‐term functional outcome in patients with hypertensive intracerebral hemorrhage (HICH) without chronic kidney disease (CKD). A total of 555 patients with HICH were consecutively recruited after admission and were followed‐up for 3 months after admission. The primary outcome was poor functional outcome (modified Rankin Scale [mRS] score ≥ 3). The median serum CysC level in our cohort was 1.03 mg/L (interquartile range, .89–1.20). Patients were categorized into four groups according to the serum CysC quartiles. Multivariate logistic regression analysis revealed a negative association between serum CysC and poor functional outcome at 3‐month follow‐up (quartile [Q]1 vs. Q4: adjusted odds ratio [OR] = .260, 95% confidence interval [CI] = .098, .691, *p* < .001). The negative association between serum CysC and poor functional outcome at 3 months was more pronounced in subgroups with smaller hematoma volume (≤ 30 mL), and absence of secondary intraventricular hemorrhage (IVH). Addition of serum CysC to a model containing conventional risk factors improved the model performance with net reclassification index (NRI) of .426% (*p* < .001) and integrated discrimination improvement (IDI) of .043% (*p* < .001) for poor functional outcome. Serum CysC was found to be a negative predictor of poor short‐term functional outcome in HICH patients independent of renal function.

## 1 INTRODUCTION

Spontaneous intracerebral hemorrhage accounts for only 10%–15% of all stroke subtypes,^1^, ^2^ but is associated with disproportionately high rates of mortality and disability, especially in China.^3^ Essential hypertension is the leading cause of spontaneous intracerebral hemorrhage.^4^ Several factors, such as excessively high initial systolic blood pressure (SBP), elevated homocysteine Levels, and neutrophil to lymphocyte ratio at admission, have been shown to be related with poor prognosis of hypertensive intracerebral hemorrhage (HICH);^4^, ^5^, ^6^ however, further studies are required to identify other prognostically relevant factors.

Cystatin C (CysC) is a 120 amino‐acid protein encoded by the *Cst3* gene and is a member of the cystatin type 2 superfamily that is produced by all nucleated human cells.^7^ Serum CysC is generally considered an alternative marker of renal function. It was shown to be superior to serum creatinine (sCr) as a biomarker of early kidney dysfunction in clinical settings.^8^, ^9^ In addition, serum CysC was shown to be a stronger predictor of the risk of cardiovascular and cerebrovascular events in older persons compared to sCr.^10^, ^11^, ^12^ Serum CysC also showed an independent association with cerebral artery stenosis and mortality in patients with stroke or cardiovascular disease who did not have chronic kidney disease (CKD).^13^, ^14^, ^15^ Thus, serum CysC may have other roles in addition to its use as a marker of kidney dysfunction in patients with HICH.

The prognostic relevance of serum CysC in HICH warrants attention; however, the results of previous studies have been inconsistent. In a study, elevated serum CysC level was found to be associated with high all‐cause mortality at 5‐year follow‐up in patients with ischemic and hemorrhagic stroke.^15^ Moreover, higher serum CysC was found to predict post‐stroke fatigue in patients with HICH.^16^ However, in another study, serum CysC level exhibited a U‐shaped correlation with poor clinical outcome of stroke at 1‐year follow‐up.^17^ In another study, serum CysC level showed a negative correlation with post‐stroke cognitive dysfunction.^18^ Previous studies have generally focused on the relationship between ischemic stroke and serum CysC, while the association between serum CysC and functional outcome in HICH patients is not well characterized.

Thus, we conducted an observational study to investigate the association between serum CysC level and 3‐month functional outcome in HICH patients without CKD.

## 2 MATERIALS AND METHODS

### 2.1 Study population

This retrospective, observational study was approved by the Ethics Committee of the Affiliated Hospital of Guizhou Medical University. We conducted a retrospective analysis of 555 consecutive patients with HICH who had been admitted to the Emergency Neurology Department of the Affiliated Hospital of Guizhou Medical University between January 2019 and December 2020. The inclusion criteria were: (1) definitive history of hypertension (office SBP ≥ 140 mmHg and/or diastolic blood pressure [DBP] ≥ 90 mmHg;^19^ (2) typical bleeding sites of HICH including the basal ganglia area, thalamus, brainstem, cerebrum, and cerebellum, as confirmed by brain computed tomography (CT) or magnetic resonance imagining; (3) admission within 3 days after the onset of stroke symptoms; (4) estimated glomerular filtration rate (eGFR) ≥ 60 mL/min/1.73 m2, with the eGFR calculated according to the Modification of Diet in Renal Disease (MDRD)^20^; and (5) age ≥ 18 years. The exclusion criteria were: (1) any other type of stroke; (2) severe systemic comorbidities, such as tumors, trauma, or infectious, endocrine, hematological, or metabolic diseases, except for diabetes mellitus (DM); (3) severe chronic diseases, such as thyroid disease or secondary hypertension; (4) severe organic brain disorders.

### 2.2 Determination of clinical and biochemical indicators

The following clinical and demographic data were retrieved from the electronic medical records: (1) sex; (2) age at diagnosis; (3) height; (4) weight; (5) history of smoking and consumption of alcohol; (6) SBP and DBP at admission; and (7) co‐morbidities, including hypertension, DM, hemorrhage, and infarction. The bleeding location, time from onset to admission, Glasgow Coma Scale (GCS) score at admission, and National Institutes of Health Stroke Scale (NIHSS) score at admission. Body mass index (BMI) was calculated using the formula: BMI = weight/height.^2^ Mean arterial pressure (MAP) was calculated according to admission SBP and DBP (MAP = DBP + 1/3[SBP‐DBP]). Serial venous blood samples were obtained on admission (before any therapeutic interventions) and serum CysC level was measured using a particle‐enhanced immunonephelometric assay (Hitachi High‐Technologies, Tokyo, Japan), with a serum CysC reference range of .59–1.03 mg/L, according to the Guizhou Medical University protocol. Other laboratory parameters at admission, including blood glucose, total cholesterol (TC), triglycerides (TG), high‐density lipoprotein (HDL), low‐density lipoprotein (LDL), sCr, blood uric acid (UA), and blood urea nitrogen (BUN) were also recorded.

### 2.3 Management of patients with HICH

We follow the American Stroke Association^21^ and European Stroke Organisation^22^ recommendations for the treatment of non‐traumatic spontaneous intracerebral hemorrhage. For patients who met the indications for neurosurgical intervention, we actively adopted corresponding surgery (minimally invasive surgery or craniotomy evacuation of hematoma) with the consent of the patients and/or their families. Standard conservative medical care according to guidelines was provided for patients who did not require or refused surgery. The presence or absence of secondary intraventricular hemorrhage (IVH) and hydrocephalus was noted. Two parameters related to in‐hospital treatment (whether external ventricular drain placement or surgical hematoma evacuation was undertaken) and the total length of hospital stay were also recorded.

### 2.4 Scale measurement

The functional outcome was measured using the modified Rankin Scale (mRS) at the 3‐month follow‐up. Patients with mRS score ≥ 3 were defined as having a poor functional outcome, as per the criteria used in a previous study.^23^ The intracerebral hemorrhage volume was assessed upon admission with the ABC/2 method as follows: (1) A = greatest hemorrhage diameter by CT; (2) B = diameter perpendicular to A; and (3) C = the approximate number of CT slices with hemorrhage multiplied by the slice thickness. The eGFR was calculated using the MDRD guidelines^20^ as follows: eGFR (mL/min/1.73 m2) = 175 × SCr (mg/dL)^−1.154^ × age (years)^−.203^ × 1.212 (if African‐American) × .742 (if female). All measurements were independently performed by two researchers who were blinded to the laboratory results.

### 2.5 Statistical analysis

Data analyses were conducted using the SPSS version 23.0 software. Study population was first divided into four groups based on the serum CysC quartiles (quartile [Q]1 < .89 mg/L, Q2 = .89–1.03 mg/L, Q3 = 1.03–1.20 mg/L, Q4 > 1.20 mg/L). Continuous variables were expressed as mean ± standard deviation for normally distributed data and as median and interquartile range for non‐normally distributed data. Continuous variables were compared using ANOVA or the Kruskal–Wallis test, as appropriate. Categorical variables were expressed as frequency (percent) and were compared using the Chi‐squared test.

Using functional outcome at 3‐month follow‐up as the dependent variable, univariate logistic regression analysis was performed between the 3‐month functional outcome and clinical parameters. Multiple logistic regression analyses were conducted to explore the association of serum CysC and 3‐month poor functional outcome; variables with *p*‐values < .05 in univariate analysis were adjusted in the multivariate model. Model 1 was adjusted for sex, age, blood glucose, SBP, MAP, admission GCS score, admission NIHSS score, hematoma volume, brainstem hemorrhage, basal ganglia hemorrhage, time to admission, presence or absence of IVH, length of hospital stay, surgical interventions or not. Model 2 was further adjusted for DBP and HDL. Model 3 was additionally adjusted for eGFR. The correlation between serum CysC and 3‐month poor functional outcome in HICH patients was further evaluated using a logistic regression model with restricted cubic splines. Besides, subgroup analyses were also performed according to sex, age (≥65 years; <65 years), hematoma volume (≤30 mL; >30 mL), and presence or absence of IVH. Integrated discrimination improvement (IDI) and net reclassification index (NRI) were used to assess improvement in model performance by adding serum CysC to the conventional model (risk factors in model 3 and eGFR) to assess the incremental value of serum CysC in risk prediction for the 3‐month functional outcome independent of renal function. *p*‐values < .05 were considered indicative of statistical significance.

## 3 RESULTS

### 3.1 Baseline characteristics of the study population

**FIGURE 1:**
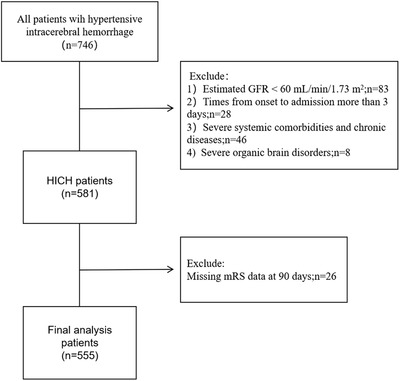
Flowchart showing the selection of the study population

A total of 555 patients with HICH were enrolled. The flowchart of participants selection is shown in Figure 1. The mean age of the included patients was 58.3 ± 11.7 years, and 377 (67.9%) were male. The median serum CysC in our cohort was 1.03 mg/L (interquartile range, .89–1.20 mg/L). The study population was categorized into four groups according to the serum CysC quartiles: 126 patients were in Q1, 147 were in Q2, 144 were in Q3, and 138 were in Q4. The baseline characteristics of the study population are summarized in Table 1. As compared with patients with lower serum CysC, those with higher serum CysC were more likely to be older, male, and have a history of smoking, with a lower blood glucose and eGFR, and higher BUN, UA, and sCr. They also had smaller hematoma volume. Patients with higher serum CysC were more likely to require surgical intervention. There were no significant differences between the four groups with respect to the other baseline characteristics.

**TABLE 1:** Baseline characteristics of patients with hypertensive intracerebral hemorrhage according to serum CysC quartiles

	Admission serum CysC, mg/L				
Variables	Q1 (<.89) (*n* = 126)	Q2 (.89–1.03) (*n* = 147)	Q3 (1.03–1.20) (*n* = 144)	Q4 (>1.20) (*n* = 138)	*p*‐value
Age (years)	54.6 ± 10.3	57.5 ± 10.9	58.9 ± 12.1	62.0 ± 12.4	<.001
Male sex, *n* (%)	62 (49.2)	97 (65.9)	104 (75.2)	114 (82.6)	<.001
Smoking, *n* (%)	38 (30.1)	47 (31.9)	69 (47.9)	73 (52.8)	<.001
Drinking, *n* (%)	26 (20.6)	36 (24.4)	33 (22.9)	46 (33.3)	.085
Diabetes, *n* (%)	7 (5.5)	9 (6.1)	13 (9.0)	16 (11.5)	.231
BMI (kg/m^2^)	23.8 (21.4, 27.1)	23.9 (21.3, 26.6)	24.5 (22.0, 26.8)	25 (21.5, 27.4)	.265
SBP (mmHg)	168.5 (146.7, 191.2)	172.0 (151.0, 189.0)	168.5 (141.2, 189.7)	173.0 (149.7, 197.2)	.833
DBP (mmHg)	99.0 (86.0, 111.0)	100.0 (90.0, 111.0)	100.5 (83.2, 117.0)	101.0 (89.0, 115.2)	.629
MAP (mmHg)	122.6 (106.6, 139.7)	125 (110, 138.6)	124 (106.25, 142.1)	125.3 (110.1, 140.0)	.749
BUN (mmol/L)	4.1 (3.3, 4.9)	4.5 (3.6, 5.5)	4.6 (4.0, 5.7)	5.4 (4.5, 6.7)	<.001
sCr (μmol/L)	51.7 (46.9, 61.0)	61.4 (52.0, 71.7)	67.0 (57.0, 81.0)	82.9 (68.9, 95.8)	<.001
eGFR (mL/min/1.73 m^2^)	125.5 (110.9, 152.1)	113.5 (97.5, 128.2)	99.9 (86.1, 118.4)	83.6 (71.4, 100.27)	<.001
UA (μmol/L)	282.4 (235.1, 351.7)	317.2 (245.8, 388.0)	331.1 (279.7, 400.8)	391.5 (317.8, 469.6)	<.001
Glucose (mmol/L)	7.5 (6.4, 9.3)	7.37 (6.3, 8.6)	7.2 (6.1, 9.3)	6.6 (5.7, 8.5)	.021
TG (mmol/L)	1.2 (.8, 1.7)	1.2 (.9, 1.8)	1.3 (.8, 1.7)	1.1 (.83, 1.6)	.593
TC (mmol/L)	4.2 (3.4, 4.8)	4.4 (3.8, 4.9)	4.1 (3.5, 4.8)	4.2 (3.6, 4.7)	.184
HDL (mmol/L)	1.2 (.9, 1.5)	1.1 (.9, 1.4)	1.2 (.9, 1.5)	1.2 (1.0, 1.4)	.790
LDL (mmol/L)	2.5 (1.9, 3.0)	2.7 (2.2, 3.2)	2.5 (2.0, 3.2)	2.6 (1.9, 3.1)	.126
Admission GCS score	10.5 (7.0, 14.0)	12.0 (8.0, 14.0)	12.0 (7.2, 15.0)	13.0 (9.0, 15.0)	.185
Admission NIHSS	16.0 (8.7, 23.0)	13.0 (8.0, 22.0)	14.5 (5.0, 20.0)	13.0 (6.0, 20.0)	.290
Time to admission (*h*)	4.0 (3.0, 10.0)	5.0 (2.0, 10.0)	4.2 (2.0, 10.0)	5.0 (2.0, 15.0)	.828
Hematoma volume (mL)	20.5 (8.0, 40.0)	17.0 (8.0, 35.0)	12.1 (6.0, 26.9)	13.4 (5.0, 35.0)	.005
Hematoma, categorical					
≤30 mL, *n* (%)	80 (63.5)	101 (68.7)	115 (79.8)	101 (73.1)	.022
>30 mL, *n* (%)	46 (36.5)	46 (31.2)	29 (20.2)	37 (26.9)	
Hematoma location					
Basal ganglia, *n* (%)	67 (53.1)	91 (61.9)	88 (61.1)	73 (52.8)	.251
Brainstem, *n* (%)	10 (7.9)	8 (5.4)	13 (9.0)	12 (8.6)	.659
Thalamus, *n* (%)	18 (14.2)	22 (14.9)	15 (10.4)	30 (21.7)	.067
Cerebellum, *n* (%)	13 (10.3)	12 (8.1)	10 (6.9)	10 (7.2)	.747
Brain lobe, *n* (%)	43 (34.1)	42 (28.5)	35 (24.3)	34 (24.6)	.248
IVH, *n* (%)	61 (48.4)	66 (44.8)	64 (44.4)	65 (47.1)	.903
Surgical interventions, *n* (%)	77 (61.1)	77 (52.3)	68 (47.2)	55 (39.8)	.005
Hydrocephalus, *n* (%)	3 (2.3)	5 (3.4)	7 (4.8)	5 (3.6)	.749
EVD, *n* (%)	3 (2.3)	5 (3.4)	4 (2.7)	4 (2.8)	.967
Length of hospital stay	11 (9, 14.2)	12 (9, 15)	11 (8, 13)	11 (8.7, 15)	.095

*Note*: Data presented as mean ± SD, *n* (%), or median (interquartile range [IQR]) unless otherwise noted.

Abbreviations: BMI, body mass index; BUN, blood urea nitrogen; CysC, cystatin C; DBP, diastolic blood pressure; eGFR, estimated glomerular filtration rate; EVD, external ventricular drain; GCS, Glasgow Coma Scale; HDL, high‐density lipoprotein; IVH, intraventricular hemorrhage; LDL, low‐density lipoprotein; MAP, mean arterial pressure; NIHSS, National Institutes of Health Stroke Scale; SBP, systolic blood pressure; sCr, serum creatinine; TC, total serum cholesterol; TG, triglycerides; UA, uric acid.

### 3.2 Univariate logistic analysis of risk factors for 3‐month functional outcome

At 3‐month follow‐up, 343 patients (mean age: 58.3 ± 11.7 years; 232 [67.6%] males) were in the good functional outcome subgroup and 212 patients (58.4 ± 11.8 years; 145 [68.4%] male) were in the poor functional outcome subgroup (mRS score ≥ 3). To analyze the association between risk factors and functional outcome in patients with HICH, we used poor functional outcome as a dependent variable for univariate logistic analysis. As shown in Table S1, SBP, DBP, MAP, blood glucose, HDL, admission NIHSS score, hematoma volume, proportion of patients with surgical intervention, presence of IVH, length of hospital stay, and basal ganglia and brainstem hemorrhage were significantly higher in the poor functional outcome subgroup, while the time to admission and admission GCS score were significantly lower.

### 3.3 Relationship between serum CysC and 3‐month functional outcome

**FIGURE 2:**
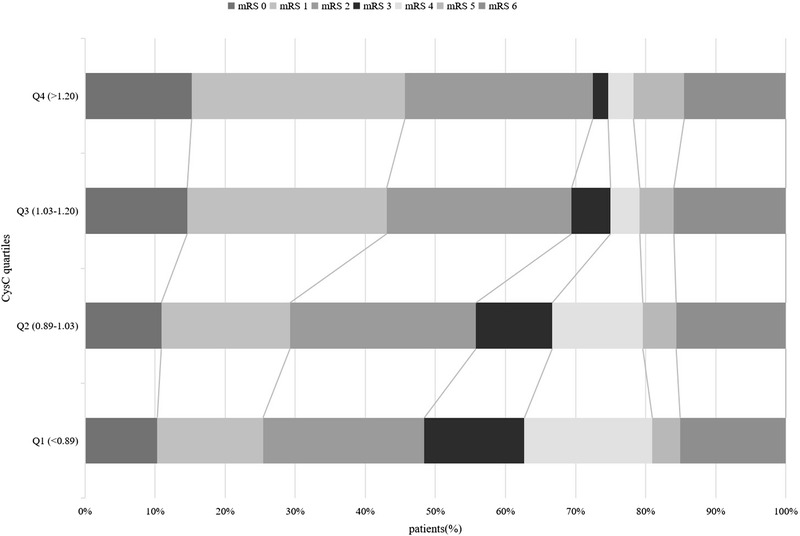
Relationship between mRS score and serum CysC level

**FIGURE 3:**
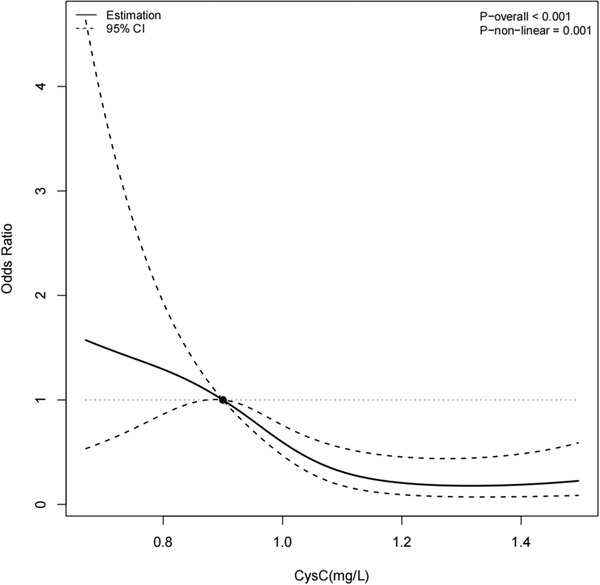
Correlation of serum CysC with poor functional outcome. We used restricted cubic spline regression to predict the odds ratio and 95% confidence intervals based on the serum CysC levels adjusted by variables in model 3 for patients with HICH

Figure 2 shows the distribution of 3‐month mRS score and serum CysC quartiles among all patients with HICH. A negative stepwise, dose‐dependent relationship between serum CysC level and 3‐month functional outcome was observed (*p* = .003). The results of logistic regression analysis showing association between serum CysC and 3‐month functional outcome are presented in Table 2. Compared to the lowest serum CysC quartiles, the odds ratio (OR) of 3‐month poor functional outcome with the highest serum CysC quartiles was .357 (95% confidence interval [CI] = .214, .595, *P*_trend_ < .001); after adjusting for potential confounders in models 1, 2, and 3, the ORs were .257 (95% CI = .111, .595, *P*_trend_ < .001), .259 (95% CI = .112, .600, *P*_trend_ < .001), and .260 (95% CI = .098, .691, *P*_trend_ < .001), respectively. A negative correlation was observed between serum CysC and 3‐month functional outcome in all patients with HICH according to logistic regression model with restricted cubic splines analysis (Figure 3).

**TABLE 2:** Odds ratios (ORs) and 95% confidence intervals (CIs) for the association between serum CysC quartiles and risk of poor functional outcome in patients with hypertensive intracerebral hemorrhage

Variables	Q1 (<.89)	Q2 (.89–1.03)	Q3 (1.03–1.20)	Q4 (>1.20)	*p*‐value
Unadjusted	1.000 (ref)	.774 (.461, 1.199)	.413 (.251, .679)	.357 (.214, .595)	<.001
Model 1	1.000 (ref)	.963 (.472, 1.964)	.273 (.123, .603)	.257 (.111, .595)	<.001
Model 2	1.000 (ref)	.989 (.483, 2.018)	.277 (.125, .613)	.259 (.112, .600)	<.001
Model 3	1.000 (ref)	.982 (.475, 2.027)	.271 (.118, .619)	.260 (.098, .691)	.001

*Note*: Model 1: Adjusted for sex, age, glucose, systolic blood pressure, mean arterial pressure, admission Glasgow Coma Scale score, Admission National Institutes of Health Stroke Scale score, hematoma volume, brainstem hemorrhage, basal ganglia hemorrhage, time to admission, intraventricular hemorrhage, length of hospital stay, surgical interventions. Model 2: Adjusted for model 1 and further adjusted for diastolic blood pressure and high‐density lipoprotein. Model 3: Adjusted for model 2 and further adjusted for estimated glomerular filtration rate.

### 3.4 Subgroup analysis for association between serum CysC and poor functional outcome

To further explore the association between serum CysC and 3‐month poor functional outcome in different subgroups, we grouped the patients according to sex, age, hematoma volume, presence or absence of IVH, and analyzed the outcomes according to serum CysC quartiles after adjusting for parameters in model 3. As shown in Table S2, there was no heterogeneity in the effects of serum CysC on the functional outcome subgroups classified by sex or age. Of note, the trend of negative association between serum CysC and poor functional outcome was more pronounced in the subgroups with smaller hematoma volume (≤30 mL) and absence of IVH. There were no significant interaction between serum CysC and these parameters.

### 3.5 Reclassification value of serum CysC

NRI and IDI values showing the incremental value of serum CysC in risk prediction for the functional outcome are presented in Table 3. In all patients with HICH, after adding serum CysC to a basic model with important prognostic factors (risk factors in model 3 and eGFR), the risk reclassification appeared to be significant (NRI .426%, *p* < .001; IDI .043%, *p* < .001), and the risk reclassification still appeared to be significant in patients with smaller hematoma volume (≤30 mL) (NRI .447%, *p* < .001; IDI .053%, *p* < .001) and absence of IVH (NRI .553%, *p* < .001; IDI .062%, *p* < .001).

**TABLE 3:** Reclassification and Discrimination statistics for outcome by serum CysC in HICH patients

Variables	NRI (continuous)		IDI	
	Estimate (95% CI), %	*p*‐value	Estimate (95% CI), %	*p*‐value
**Total patient**				
Conventional model	1.000 (ref)		1.000 (ref)	
Conventional model+CysC	.426 (.258, .593)	<.001	.043 (.026, .059)	<.001
**Hematoma volume ≤30mL**				
Conventional model	1.000 (ref)		1.000 (ref)	
Conventional model + CysC	.447 (.232, .662)	<.001	.053 (.030, .077)	<.001
**Absence of IVH**				
Conventional model	1.000 (ref)		1.000 (ref)	
Conventional model + CysC	.553 (.304, .802)	<.001	.062 (.028, .095)	<.001

*Note*: The conventional model included systolic blood pressure, admission National Institutes of Health Stroke Scale score, brainstem hemorrhage, basal ganglia hemorrhage, intraventricular hemorrhage, estimated glomerular filtration rate.

Abbreviations: CysC, cystatin C; HICH, hypertensive intracerebral hemorrhage; CI, confidence interval; IDI, integrated discrimination index; IVH, intraventricular hemorrhage; NRI, net reclassification improvement.

## 4 DISCUSSION

CysC, an endogenous inhibitor of cysteine proteinase, is highly associated with cerebrovascular events.^12^, ^24^, ^25^ There are several key findings of the present study. Firstly, serum CysC level showed a negative association with poor functional outcome in patients with HICH independent of chronic kidney function (eGFR < 60 mL/min/1.73 m2). Secondly, adding serum CysC to conventional risk factors was found to improve risk prediction for functional outcome. Thirdly, the negative association between serum CysC and poor the functional outcome was more pronounced in patients with smaller hematoma volume and those who did not have IVH.

As impaired renal function is an important poor prognostic factor in patients with intracerebral hemorrhage,^26^ to rule out renal insufficiency, we excluded patients with eGFR < 60 mL/min/1.73 m2 from the analysis.^27^, ^28^ On examining the relationship between serum CysC and various clinical parameters in patients with HICH, besides its association with renal function, higher serum CysC quartiles were observed in males and elderly patients, which is consistent with the findings of previous studies.^29^, ^30^ Moreover, higher serum CysC quartiles were associated with smaller hematoma volume, which is at odds with the findings of a previous study. In the study by Xiao et al.,^31^ larger hematoma volume was associated with higher serum CysC level. There may be two potential reasons for this difference. The first reason is the differences with respect to the study population. Xiao et al. did not exclude patients with CKD, and on further analysis they found that their result was precisely because patients with severe atherosclerosis often have both severe renal insufficiency and cerebrovascular disease; therefore, high levels of CysC showed a positive correlation with the hematoma volume. However, we eliminated this potential confounding influence by excluding patients with eGFR < 60 mL/min/1.73 m2. The second reason is the small sample size in their study (*n* = 69). The relationship between hematoma volume and serum CysC observed in our study can be explained as follows. Hemorrhage stroke is caused by rupture of blood vessels in the brain,^32^ and intracerebral hemorrhage leads to injury manifested by impaired blood‐brain barrier (BBB) integrity and perihematomal edema.^33^ Serum CysC is a small molecule protein that may infiltrate into perihematomal edema via the impaired BBB in patients with HICH, and the increase in hematoma volume results in entry of more CysC in the brain tissue. This may also explain the association of serum CysC with hematoma volume, but not with the site of hemorrhage.

Most previous studies investigating the prognostic significance of serum CysC in cardiovascular and cerebrovascular diseases have demonstrated poor prognostic value of high serum CysC level;^15^, ^16^, ^34^, ^35^ however, these previous studies generally did not exclude patients with CKD, and reduced kidney function itself is associated with poor clinical outcomes in patients with ICH. In a study, reduced eGFR was found to be associated with an increased risk of poor functional outcomes at 3‐month follow‐up in patients with ICH, but high serum CysC was not.^36^ Another study observed a U‐shaped association between serum CysC and poor functional outcome in acute ischemic stroke.^17^ Guo et al. reported a negative linear dose‐response correlation between CysC and cognitive dysfunction in ischemic stroke patients with normal renal function, but not in those with abnormal renal function,^18^ demonstrating that serum CysC plays a unique role in stroke patients which is independent of renal function. In our study, serum CysC levels showed a negative association with short‐term functional outcome in non‐CKD patients with HICH. On multivariate logistic regression analysis, lower serum CysC was associated with poor functional outcome even after adjusting for various potential confounding factors. Furthermore, adding serum CysC to a model containing conventional risk factors, including SBP, admission NHISS score, brainstem hemorrhage, basal ganglia hemorrhage, presence of IVH, surgical interventions, improved the model performance with IDI and NRI for poor functional outcome.

Several potential pathophysiological pathways can explain the mechanisms underlying the association of increased serum CysC level with decreased risk of poor short‐term functional outcome in non‐CKD patients with HICH. CysC is more abundant in the central nervous system, especially in the cerebrospinal fluid.^37^ Studies suggest that the severity of ICH‐induced neural damage is exacerbated by infiltration of T‐cells, monocytes, and especially neutrophils into the perihematomal edema.^38^, ^39^ CysC, also known as neutrophil growth factor, affects the migration and phagocytosis of neutrophils and participates in inflammation.^34^ Studies have demonstrated that CysC may exert its neuroprotective effect by preserving lysosomal integrity, which can improve BBB integrity^40^ and counteract inflammation.^34^ Moreover, the intracellular transport of CysC, as well as CysC secretion, can be modulated by neuroglobin to prevent oxidative stress‐induced neuronal death,^41^ which may be of great value for HICH treatment. Hence, we speculate that elevated CysC level around the hematoma has an effect on patient outcome and may also induce good outcomes due to the neuroprotective effects of CysC.

We further found that the negative correlation between serum CysC and 3‐month poor functional outcome was more pronounced in the setting of smaller hematoma volume and no IVH. Usually, larger hematoma volume is an important factor for severe ICH,^42^ and these patients typically require more interventions, including surgery. Therefore, various other factors may affect the prognosis of these patients. In a study, cerebrospinal fluid CysC levels were found to be significantly higher in patients with severe aneurysmal subarachnoid hemorrhage.^43^ In patients with HICH who have secondary IVH, serum CysC concentrations may be further affected because more of it may infiltrate into ventricle from the blood vessels. Serum CysC levels showed limited prognostic value in patients with HICH in these subgroups.

Some limitations of our study should be considered while interpreting the results. First, this was a retrospective observational study which does not permit any causal inferences pertaining to the relationship between serum CysC levels and HICH outcomes. Second, HICH is caused by various factors that synergistically affect various metabolic pathways, and the specific role of serum CysC in this process must be further explored. Third, only baseline serum CysC level and short‐term functional outcome were analyzed in this study. Therefore, we were unable to examine the prognostic significance of changes in CysC level and the association of CysC with long‐term functional outcome. Therefore, large‐scale, multi‐center, prospective studies with repeated measurement are required to further characterize the relationship between CysC and HICH.

## 5 CONCLUSIONS

Our findings provide new insights into the prognostic significance of serum CysC in patients with HICH. In this study, serum CysC level showed a positive association with favorable short‐term functional outcome in non‐CKD patients with HICH. This correlation reflects the non‐renal effects of serum CysC on intracerebral hemorrhage and suggests that the relationship between serum CysC and neurovascular risk may be independent of renal function.

## AUTHOR CONTRIBUTIONS

Wentao Dong and Weiqing Wei collected the data, Likun Wang and Siying Ren blindly analyzed the data, Yongfang Zhou made statistical analysis, and Yongfang Zhou and Guofeng Wu wrote the first draft and the revised versions of the paper, after comments and criticisms by coauthors.

## CONFLICT OF INTEREST

The authors declare that they have no potential conflict of interest.

## Supporting information

Supporting Information

Supporting Information
