# Supplementary material for: Lower serum cystatin C level predicts poor functional outcome in patients with hypertensive intracerebral hemorrhage independent of renal function
Source: J Clin Hypertens (Greenwich). 2022 Dec 22;25(1):86–94. doi: 10.1111/jch.14609 (PMC9832235; doi:10.1111/jch.14609)
Supplement: Supplementary file 1 — Supporting Information [file JCH-25-86-s001.docx]

**Supplementary TABLE 1** Univariate logistic analysis showing clinical/laboratory parameters associated with the risk of poor functional outcome (mRS≥3).

| **Variables** | **OR (95% CI)** | ***P* value** | **Variables** | **OR (95% CI)** | ***P* value** |
| --- | --- | --- | --- | --- | --- |
| Age | 1.000(0.986, 1.015) | 0.974 | HDL | 1.573(1.014, 2.439) | 0.043 |
| Sex, male | 1.035(0.717, 1.495) | 0.853 | LDL | 0.960(0.831, 1.108) | 0.579 |
| BMI | 1.017(0.973, 1.063) | 0.451 | Admission GCS score | 0.659(0.616, 0.704) | <0.001 |
| Diabetes | 0.884(0.468, 1.669) | 0.704 | Admission NIHSS score | 1.253(1.207, 1.300) | <0.001 |
| Smoking | 0.783(0.551, 1.112) | 0.171 | Time to admission | 0.976(0.963, 0.989) | 0.001 |
| Drinking | 0.723(0.483, 1.083) | 0.116 | IVH | 3.325(2.325, 4.756) | <0.001 |
| SBP | 1.014(1.008, 1.019) | <0.001 | Surgical interventions | 5.111(3.505, 7.453) | <0.001 |
| DBP | 1.010(1.001, 1.018) | 0.024 | Hydrocephalus | 1.649(0.674, 4.030) | 0.273 |
| MAP | 1.014(1.006, 1.022) | <0.001 | EVD | 1.612(0.607, 4.443) | 0.329 |
| BUN | 0.979(0.884,1.084) | 0.682 | Hematoma volume | 1.044(1.033, 1.054) | <0.001 |
| sCr | 0.992(0.983, 1.002) | 0.103 | Length of hospitalization | 1.035 (1.015, 1.056) | 0.001 |
| GFR | 1.003(0.998, 1.007) | 0.226 | Basal ganglia | 1.565(1.101, 2.224) | 0.013 |
| UA | 1.000(0.998, 1.001) | 0.749 | Brainstem | 2.427(1.290, 4.566) | 0.006 |
| Glucose | 1.166(1.093, 1.243) | <0.001 | Thalamus | 0.973(0.604, 1.567) | 0.910 |
| TG | 0.959(0.831,1.106) | 0.563 | Cerebellum | 0.715(0.371, 1.378) | 0.317 |
| TC | 1.090(0.906, 1.310) | 0.362 | Brain lobe | 1.360(0.940, 1.984) | 0.111 |

Abbreviations: BMI, body mass index; BUN, blood urea nitrogen; DBP, diastolic blood pressure; eGFR, estimated glomerular filtration rate; EVD, external ventricular drain; GCS, Glasgow Coma Scale; HDL, high-density lipoprotein; HICH, hypertensive intracerebral hemorrhage; IVH, intraventricular hemorrhage; LDL, low-density lipoprotein; MAP, mean blood pressure; mRS, the modified Rankin Scale; NIHSS, National Institutes of Health Stroke Scale; SBP, systolic blood pressure; sCr, serum creatinine; TC, total serum cholesterol; TG, triglycerides; UA, uric acid.
