# Supplementary material for: Lower serum cystatin C level predicts poor functional outcome in patients with hypertensive intracerebral hemorrhage independent of renal function
Source: J Clin Hypertens (Greenwich). 2022 Dec 22;25(1):86–94. doi: 10.1111/jch.14609 (PMC9832235; doi:10.1111/jch.14609)
Supplement: Supplementary file 2 — Supporting Information [file JCH-25-86-s002.docx]

**Supplementary TABLE 2** Poor outcome according to serum CysC quartiles in subgroups

| **Subgroups** | **N** | **Serum CysC, mg/L** | **Adjusted OR (95%CI)** | ***P* value** | ***P* interaction** |
| --- | --- | --- | --- | --- | --- |
| **Sex** 0.772 | | | | | |
| Male | 62 | Q1 (<0.89) | 1.000(ref) |  |  |
|  | 97 | Q2 (0.89-1.03) | 0.417(0.143, 1.217) | 0.110 |  |
|  | 104 | Q3 (1.03-1.20) | 0.130(0.028, 0.601) | 0.009 |  |
|  | 114 | Q4 (>1.20) | 0.151(0.025, 0.922) | 0.041 |  |
| Female | 64 | Q1 (<0.89) | 1.000(ref) |  |  |
|  | 50 | Q2 (0.89-1.03) | 0.420(0.144, 1.219) | 0.111 |  |
|  | 40 | Q3 (1.03-1.20) | 0.131(0.029, 0.593) | 0.008 |  |
|  | 24 | Q4 (>1.20) | 0.152(0.026, 0.887) | 0.036 |  |
| **Age (years)** 0.115 | | | | | |
| ≥65 | 61 | Q1 (<0.89) | 1.000(ref) |  |  |
|  | 38 | Q2 (0.89-1.03) | 2.50(0.295, 21.178) | 0.401 |  |
|  | 49 | Q3 (1.03-1.20) | 0.063(0.006, 0.667) | 0.021 |  |
|  | 64 | Q4 (>1.20) | 0.058(0.005, 0.667) | 0.022 |  |
| <65 | 105 | Q1 (<0.89) | 1.000(ref) |  |  |
|  | 109 | Q2 (0.89-1.03) | 0.949(0.411, 2.190) | 0.902 |  |
|  | 95 | Q3 (1.03-1.20) | 0.305(0.118, 0.790) | 0.014 |  |
|  | 74 | Q4 (>1.20) | 0.279(0.091, 0.833) | 0.022 |  |
| **Hematoma volume (mL)**  0.282 | | | | | |
| ≤30 | 80 | Q1 (<0.89) | 1.000(ref) |  |  |
|  | 101 | Q2 (0.89-1.03) | 1.106(0.462, 2.650) | 0.806 |  |
|  | 115 | Q3 (1.03-1.20) | 0.292(0.110, 0.774) | 0.015 |  |
|  | 101 | Q4 (>1.20) | 0.242(0.080, 0.736) | 0.013 |  |
| >30 | 46 | Q1 (<0.89) | 1.000(ref) |  |  |
|  | 46 | Q2 (0.89-1.03) | 0.865(0.159, 4.701) | 0.866 |  |
|  | 29 | Q3 (1.03-1.20) | 0.153(0.020, 1.187) | 0.073 |  |
|  | 37 | Q4 (>1.20) | 0.155(0.019, 1.239) | 0.079 |  |
| **IVH**  0.815 | | | | | |
| Yes | 61 | Q1 (<0.89) | 1.000(ref) |  |  |
|  | 66 | Q2 (0.89-1.03) | 1.056(0.346, 3.221) | 0.924 |  |
|  | 64 | Q3 (1.03-1.20) | 0.477(0.157, 1.451) | 0.192 |  |
|  | 65 | Q4 (>1.20) | 0.306(0.095, 0.988) | 0.048 |  |
| No | 65 | Q1 (<0.89) | 1.000(ref) |  |  |
|  | 81 | Q2 (0.89-1.03) | 0.864(0.322, 2.320) | 0.772 |  |
|  | 80 | Q3 (1.03-1.20) | 0.206(0.066, 0.646) | 0.007 |  |
|  | 73 | Q4 (>1.20) | 0.224(0.072, 0.696) | 0.010 |  |

Abbreviations: CysC, cystatin C; IVH, intraventricular hemorrhage.
